# Valorization of Chum Salmon (*Oncorhynchus keta*) Processing By-Products: High-Value Functional Food Ingredients for Skin Health

**DOI:** 10.3390/md24050178

**Published:** 2026-05-14

**Authors:** Wook-Chul Kim, Yun-Su Lee, Seo-Rin Jung, Sun Young Park, Hyun Jung Yun, Jae-Young Oh, Seung-Hong Lee

**Affiliations:** 1 Department of Medical Science, Soonchunhyang University, Asan 31538, Republic of Korea; wookchul0828@naver.com (W.-C.K.); yslee9802@naver.com (Y.-S.L.); jsrpa020329@naver.com (S.-R.J.); 2 Food Safety and Processing Research Division, National Institute of Fisheries Science, 216, Gijanghaean-ro, Gijang-eup, Busan 46083, Republic of Korea; tjsdud3591@korea.kr (S.Y.P.); yhj0412@korea.kr (H.J.Y.); 3 Aquaculture Industry Division, West Sea Fisheries Research Institute, National Institute of Fisheries Science, 14, Seonnyeobawi-ro, Jung-gu, Incheon 22383, Republic of Korea; 4 Department of Pharmaceutical Engineering, Soonchunhyang University, Asan 31538, Republic of Korea

**Keywords:** fish processing byproducts, enzyme hydrolysate, chum salmon (*Oncorhynchus keta*), functional food ingredients, skin health

## Abstract

The strategic recycling of fish processing byproducts as functional materials has attracted increasing attention for sustainable development and human health. In this study, we investigated the dermatological impact of chum salmon (*Oncorhynchus keta*) byproduct enzyme hydrolysates (OKPE) administered as a dietary supplement in mice. After eight weeks of OKPE administration, epidermal integrity was improved, as evidenced by a significant attenuation of transepidermal water loss (TEWL). These phenotypic improvements were associated with the regulation of aquaporin-mediated water transport, hyaluronan metabolism, and epidermal differentiation programs. Furthermore, OKPE intake promoted accelerated collagen biosynthesis. Amino acid profiling revealed that OKPE is uniquely enriched in residues essential for both natural moisturizing factor (NMF) synthesis and collagenous scaffold formation. Collectively, these findings suggest that OKPE has potential as a functional food ingredient for reinforcing the skin barrier and improving skin hydration.

## 1. Introduction

Fish is a major global food resource, valued for its high-quality protein content and diverse bioactive compounds. Accordingly, the global fish processing industry has expanded substantially in response to growing demand [1,2]. This expansion inevitably generates large quantities of byproducts, including heads, skin, bones, and viscera [3]. The management of these byproduct streams increases production costs and generates organic residues and wastewater, placing pressure on terrestrial and aquatic environments and raising concerns regarding the sustainability of food production systems [4]. Consequently, the development of effective strategies for fish processing byproducts has become an essential objective. Their utilizing as high-value functional ingredients is increasingly regarded as a core component of the circular bioeconomy [5,6,7].

Among commercially important fish species, salmon occupies a particularly prominent position in the global market, and its large-scale consumption inevitably results in substantial volumes of processing byproducts [8]. These byproducts are well recognized as rich protein sources; however, their functional potential remains underexplored Previous studies have shown that enzymatic hydrolysates derived from fish byproducts exhibit diverse bioactivities, including antioxidants, anti-inflammatory, and metabolic regulatory effects [9,10,11]. However, investigations into their biological activities as functional ingredients supporting skin health remain limited. the effects of salmon processing by-product hydrolysates on epidermal barrier integrity, transepidermal water loss (TEWL), and moisture-retention-associated mechanisms have not been fully elucidated.

Skin hydration plays a fundamental role in dermatological health by maintaining the structural integrity and physiological function of the stratum corneum [12]. This outer epidermal layer consists of an organized matrix of corneocytes and intercellular lipids, and its barrier function is highly dependent on adequate water content to protect against environmental stressors, including pollutants, allergens, and microbial exposure [13,14]. Disruption of skin hydration compromises barrier integrity and promotes a cycle of increased transepidermal water loss (TEWL), which further exacerbates dehydration [15]. Clinically, this condition is associated with xerosis, surface roughness, pruritus, and heightened sensitivity to external stimuli, ultimately reducing skin comfort and quality of life [16]. Therefore, maintaining optimal skin hydration is a primary objective in the preservation of skin health and overall human well-being.

Skin health management has traditionally relied on topical cosmetic and dermatological formulations as the primary means of improving skin condition. Although such approaches can effectively alleviate surface dryness, their overall efficacy is constrained by the stratum corneum’s intrinsic barrier function, which limits the penetration of active compounds into deeper skin layers [17]. Consequently, topical treatments are generally restricted to short-term surface effects and have limited capacity to modulate endogenous processes, including collagen and hyaluronan synthesis, essential for maintaining long-term skin structure and function [18]. As a result, alternative strategies for skin health management have increasingly been explored. Recently, systemic nutritional approaches have gained attention as a means of supporting skin function by delivering bioactive nutrients through circulation to affect intrinsic physiological processes within the skin [19,20].

In this study, we investigated the effects of orally administered enzymatic hydrolysates derived from chum salmon (*Oncorhynchus keta*) processing byproducts on skin barrier function and hydration. The findings provide scientific evidence supporting the use of enzymatic hydrolysates derived from *O. keta* processing byproducts as a sustainable, high-value source of functional food ingredients for maintaining skin health.

## 2. Results

### 2.1. Effects of OKPE on Body Weight and Food Intake

**Figure 1:**
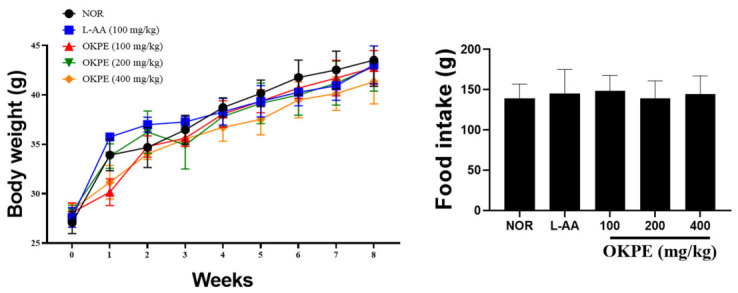
Effects of OKPE on body weight and food intake. Body weight and food intake were monitored weekly for eight weeks in mice orally administered OKPE (100, 200, or 400 mg/kg/day) or L-AA (100 mg/kg/day) as a positive control, compared with the normal control group. Data are expressed as mean ± SD (*n* = 8 per group).

To determine whether long-term oral administration of OKPE affected general growth or feeding behavior, body weight and food intake were monitored throughout the 8-week experimental period. As shown in Figure 1, mice in all experimental groups exhibited a comparable pattern of gradual weight gain over time. No abnormal deviations in body weight trajectories were observed in the OKPE-treated groups compared with the normal control group at any time point. Food intake remained stable across all groups. The average food intake values in the experimental groups ranged from 140 to 149 g, with standard deviations ranging from 17 to 30, indicating no substantial intergroup differences. Importantly, OKPE administration at doses up to 400 mg/kg/day induced no noticeable changes in daily food consumption. These findings indicate that OKPE did not affect appetite or normal growth progression, suggesting that alterations in dietary intake or body weight did not confound the observed skin-related effects.

### 2.2. OKPE Improves Skin Hydration-Related Factors

**Figure 2:**
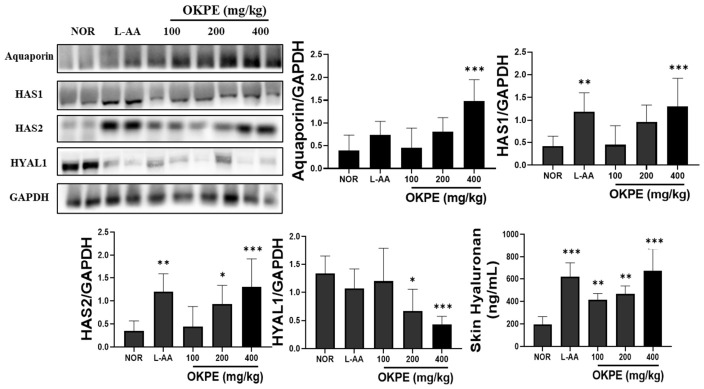
OKPE enhances skin hydration-related factors and associated protein expression. Skin hyaluronan content was quantified using ELISA in dorsal skin tissue after eight weeks of OKPE administration. Representative Western blot images of aquaporin (AQP), hyaluronan synthase 1 (HAS1), hyaluronan synthase 2 (HAS2), and hyaluronidase 1 (HYAL1) are shown, with GAPDH used as a loading control. Densitometry was normalized to GAPDH. Data are expressed as mean ± SD (*n* = 8 per group). * *p* < 0.05, ** *p* < 0.01, and *** *p* < 0.001 compared with the normal group.

To investigate the effects of OKPE on skin hydration, key hydration-related biochemical parameters and protein expression levels were examined in dorsal skin tissue. As shown in Figure 2, OKPE administration significantly increased the expression of aquaporin, a critical mediator of epidermal water transport. Additionally, OKPE treatment upregulated the protein expression of hyaluronan synthase 1 (HAS1) and hyaluronan synthase 2 (HAS2), enzymes responsible for hyaluronan biosynthesis. Modulation of hyalu-ronidase 1 (HYAL1), an enzyme involved in hyaluronan turnover, was also observed. Consistent with these molecular changes, the hyaluronan levels in the skin tissue samples were significantly increased following OKPE treatment. Collectively, these results indicate that OKPE enhances skin hydration through coordinated regulation of hydration-related molecular factors.

### 2.3. OKPE Enhances Tight Junction-Related Protein Expression in the Skin

**Figure 3:**
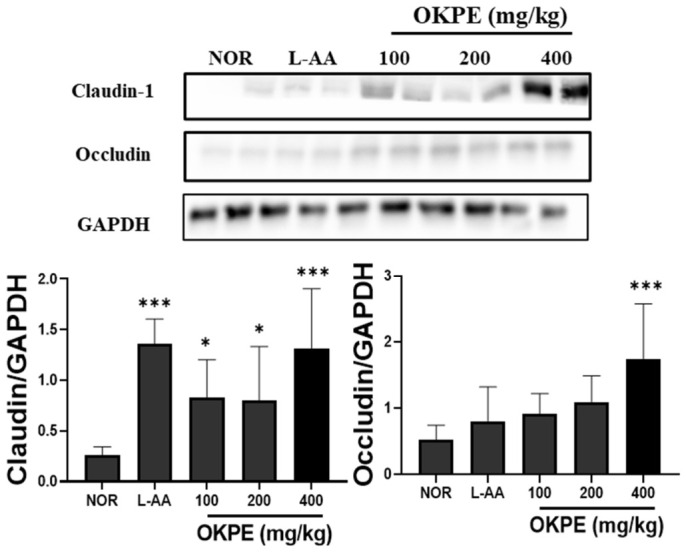
OKPE upregulates tight junction-related protein expression in skin tissue. Representative Western blot images and densitometry of tight junction-associated proteins, including claudin-1 and occludin, are shown for dorsal skin tissue. Protein expression levels were normalized to GAPDH and expressed relative to the control group. Data are presented as mean ± SD (*n* = 8 per group). * *p* < 0.05, and *** *p* < 0.001 compared with the normal group.

Previous studies have reported the importance of tight junctions in maintaining epidermal barrier integrity [21]. As shown in Figure 3, OKPE treatment increased the expression levels of claudin-1 and occludin in skin tissue compared with the control group. The upregulation of these tight junction proteins suggests that OKPE strengthens intercellular junctions within the epidermis. This enhancement of tight junction-associated proteins supports the contribution of OKPE to improved barrier integrity, complementing the observed increases in skin hydration-related parameters.

### 2.4. Effects of OKPE on Skin Components and Skin Barrier Function

**Figure 4:**
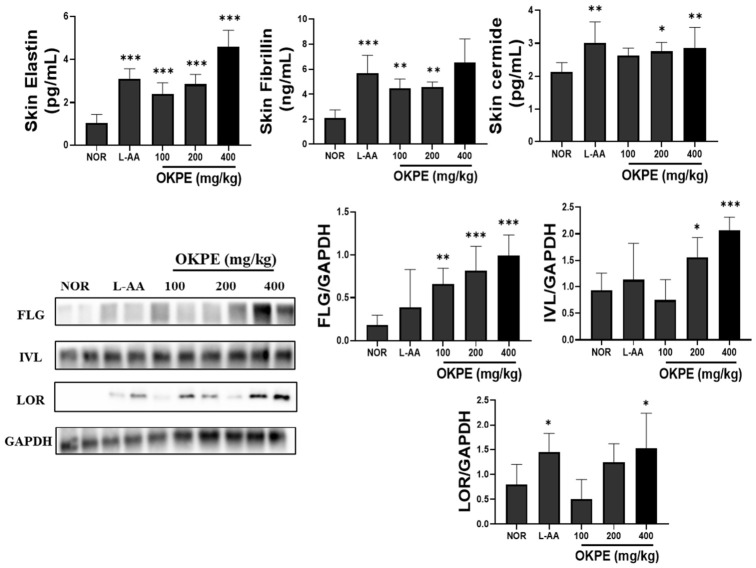
Effects of OKPE on skin components and skin barrier-related protein expression. Skin component parameters (ceramide, elastin, and fibrillin) were quantified using ELISA. Representative Western blot images of filaggrin (FLG), involucrin (IVL), and loricrin (LOR), major epidermal structural components associated with skin barrier function, with GAPDH used as a loading control. Corresponding densitometry was normalized to GAPDH. Data are presented as mean ± SD (*n* = 8 per group). * *p* < 0.05, ** *p* < 0.01, and *** *p* < 0.001 compared with the normal group.

Skin moisturization and barrier function depend on the coordinated regulation of epidermal structural proteins and moisture-retaining components. Epidermal structural proteins, including filaggrin (FLG), involucrin (IVL), and loricrin (LOR), are critical for epidermal differentiation and formation of an effective barrier, thereby contributing to water retention at the skin surface [22]. Accordingly, the effects of OKPE on epidermal barrier components were examined. As shown in Figure 4, OKPE administration significantly improved skin epidermal structural protein compared with the control group, suggesting enhanced organization of the epidermal barrier and reinforcement of the cornified envelope. Additionally, ceramides, the major lipid constituents of the stratum corneum, act synergistically with cornified envelope proteins to reduce TEWL and maintain barrier integrity [23]. Dermal extracellular matrix proteins, including elastin and fibrillin, are essential for maintaining skin elasticity and tissue structure and indirectly support sustained hydration by preserving structural stability [24]. OKPE treatment significantly increased ceramide, elastin, and fibrillin levels in skin tissue. Overall, these findings demonstrate that OKPE enhances skin barrier function through multilevel regulation of epidermal differentiation markers, lipid barrier components, and dermal structural proteins.

### 2.5. Effects of OKPE on Collagen Synthesis-Related Protein Expression

**Figure 5:**
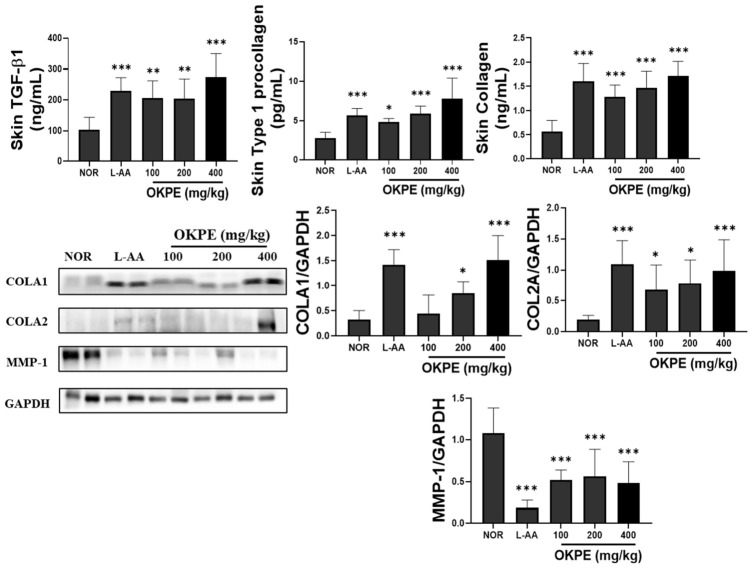
Effects of OKPE on collagen synthesis-related protein expression in skin tissue. Protein expression levels of TGF-β and type I procollagen were quantified using ELISA. Representative Western blot images and corresponding densitometry are shown. Band intensities were normalized to a loading control and expressed relative to the control group. Data are presented as mean ± SD (*n* = 8 per group). * *p* < 0.05, ** *p* < 0.01, and *** *p* < 0.001 compared with the normal group.

Skin strength and structural characteristics largely depend on the collagen-rich extracellular matrix, and reductions in collagen content are associated with skin aging [24]. This study examined whether OKPE modulates collagen homeostasis—the dynamic balance. As shown in Figure 5, the OKPE-treated groups exhibited increased TGF-β1 and procollagen type I levels, indicating upregulation of collagen biosynthetic signaling. These findings were supported by Western blotting demonstrated enhanced COL1A1 and COL1A2 expression (Figure 5). In contrast, OKPE treatment effectively downregulated the expression of matrix metalloproteinase-1 (MMP-1), the enzyme primarily responsible for collagen degradation (Figure 5). Collectively, these results suggest that OKPE promotes a favorable balance between collagen synthesis and degradation, providing a structural basis for improved skin quality and hydration.

### 2.6. Effects of OKPE on Transepidermal Water Loss and Skin Water Content

**Figure 6:**
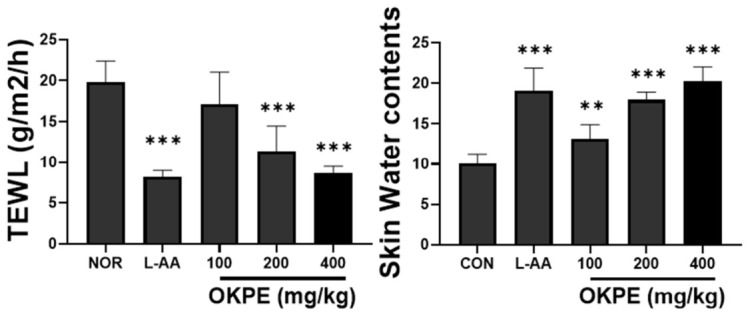
Effects of OKPE on transepidermal water loss and skin hydration. Data are presented as mean ± SD (*n* = 8 per group). ** *p* < 0.01, and *** *p* < 0.001 compared with the normal group.

TEWL and skin water content were assessed as functional indicators of epidermal barrier performance and hydration status. TEWL reflects passive water diffusion across the stratum corneum and is widely used as a sensitive marker of barrier impairment, whereas skin water content reflects the ability of the epidermis to retain moisture [25,26]. Collectively, these parameters provide insight into barrier-dependent hydration rather than transient surface moisturization. As shown in Figure 6, OKPE administration significantly decreased TEWL compared with the control group, indicating improved epidermal barrier function. Concurrently, skin water content was increased in OKPE-treated groups, suggesting enhanced moisture retention in the skin. The combined reduction in TEWL and increase in skin hydration support the conclusion that OKPE improves cutaneous moisturization through reinforcement of intrinsic barrier function rather than superficial hydration effects. Notably, more pronounced improvements in TEWL reduction and skin hydration were observed at OKPE concentrations above 200 mg/kg, suggesting that this dose range may provide useful reference information for future functional food applications targeting skin barrier support and moisturization.

### 2.7. Amino Acid Composition of OKPE

**Figure 7:**
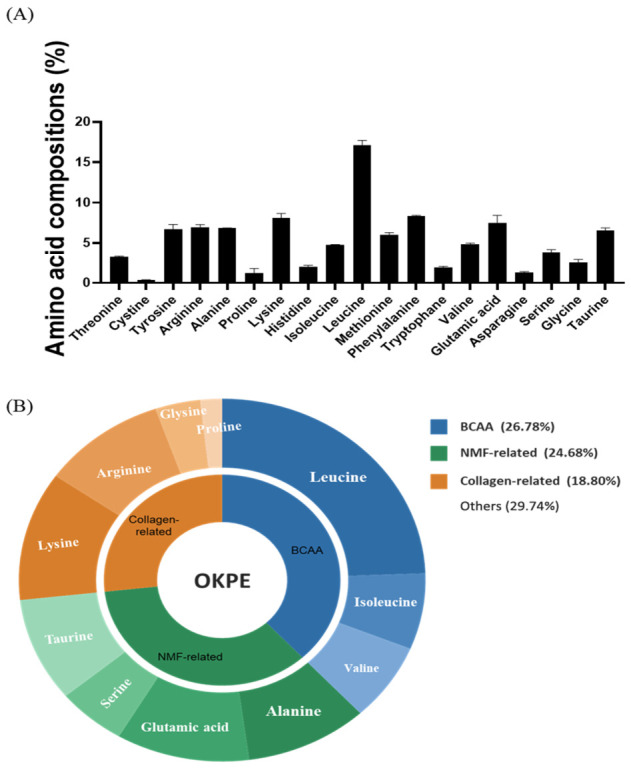
Amino acid composition of OKPE. (**A**) Percentage composition of free amino acids in OKPE. (**B**) Functional amino acid signature wheel summarizing major amino acids grouped into BCAA, NMF-related, and collagen-related categories.

To characterize amino acid composition potentially associated with the skin-related effects of OKPE and to support quality control, its free amino acid composition was analyzed. As shown in Figure 7A, arginine and leucine were consistently detected in OKPE. Notably, leucine, which has been reported to participate in epidermal hydration and barrier-related processes, was the most abundant free amino acid detected [27]. Based on their physiological relevance to skin function, amino acids identified in OKPE were categorized into branched-chain amino acids (BCAAs; leucine, isoleucine, and valine), natural moisturizing factor (NMF)-associated amino acids (alanine, glutamic acid, serine, and taurine), and collagen-related amino acids (arginine, lysine, glycine, and proline). Functional classification showed that OKPE contained BCAA (26.78%), NMF-associated amino acids (24.68%), and collagen-related amino acids (18.80%) (Figure 7B). This compositional profile provides biochemical context for the observed functional outcomes and supports the association between the free amino acid composition of OKPE and its effects on skin hydration and barrier-related parameters.

## 3. Discussion

The skin, the largest organ of the human body, functions as a dynamic barrier that regulates moisture homeostasis and provides primary protection against environmental stressors. Maintaining adequate epidermal hydration is essential for preserving epidermal architecture and barrier integrity [12,13,14]. Insufficient hydration causes adverse effects, including increased TEWL and disrupted epidermal homeostasis Clinically, these changes manifest as xerosis, pruritus, and increased sensitivity [15,16]. Persistent dehydration accelerates barrier dysfunction by impairing lipid organization and compromising the skin’s capacity for recovery and inflammatory regulation. Traditionally, strategies aimed at improving skin hydration have relied predominantly on topical cosmetic applications. While such approaches can provide temporary surface relief, their efficacy is constrained by limited penetration beyond the stratum corneum and by their transient mode of action. In contrast, emerging evidence indicates that skin health is strongly influenced by systemic physiological factors, particularly nutritional status [15,28]. This paradigm shift has driven increasing interest in dietary supplements as complementary strategies for supporting skin hydration and barrier function through intrinsic regulation rather than transient surface effects.

The utilization of fish processing byproducts, including heads, skin, bones, and fins, represents a strategic transition from waste management toward the extraction of high-value bioactive compounds for human use [5,6,7]. These byproducts serve as a concentrated source of collagenous proteins and essential amino acids that closely align with the structural and metabolic requirements of human skin [9,20]. Their valorization therefore addresses both environmental sustainability and the development of dietary supplements for skin health.

Among fish species, salmon is distinguished by its large global production scale and the continuous generation of processing byproducts. Salmon byproducts are rich in high-quality proteins and amino acids, including BCAAs associated with skin structure, hydration, and barrier-related functions. Furthermore, enzymatic hydrolysates derived from salmon have been reported to exhibit anti-inflammatory and skin-supportive effects [10]. Collectively, these characteristics support the biological suitability, industrial availability, and sustainability of salmon byproducts as functional ingredients for dietary strategies aimed at improving skin hydration and barrier function.

Accordingly, this study aimed to investigate whether oral administration of enzymatic hydrolysates of chum salmon (*O. keta*) processing byproducts (OKPE) could modulate molecular factors associated with skin hydration and barrier function and thereby support its potential as a functional ingredient.

In this study, oral administration of OKPE significantly increased skin hydration through regulation of multiple hydration-associated factors. Hyaluronic acid (HA) is a critical glycosaminoglycan with exceptional water-binding capacity and an important role in maintaining skin viscoelasticity [28,29]. Skin HA homeostasis is regulated by the balance between HAS-mediated synthesis and HYAL1-mediated degradation [30], and coordinated regulation of these pathways is essential for maintaining epidermal hydration. The results showed that OKPE treatment upregulated HAS1 and HAS2 expression while downregulating HYAL1 expression, resulting in elevated HA levels in skin tissue. Furthermore, OKPE treatment increased the expression of aquaporins (AQPs), particularly AQP3, which facilitate the transport of water and glycerol across keratinocytes. The upregulation of AQPs enhances epidermal water transport and hydration maintenance [31,32]. Collectively, these findings suggest that OKPE improves skin hydration through coordinated regulation of HA metabolism and epidermal water transport pathways.

Epidermal barrier integrity is a critical determinant of skin hydration and is formed through keratinocyte differentiation and stratum corneum formation. Key structural proteins, including FLG, IVL, and LOR, are essential for this process [22,33]. IVL and LOR are major components of the cornified envelope, which contributes to epidermal barrier stability and water retention in the stratum corneum. In the present study, OKPE administration significantly increased the expression of these differentiation-related proteins, suggesting that OKPE promotes the maturation of the epidermal layer. Filaggrin serves as a structural scaffold for keratin filaments and later degrades into NMFs. These NMFs, including PCA and various free amino acids, are fundamental to the biochemical entrapment of water molecules, thereby preserving the hydration status of the outermost epidermal barrier [34,35]. Therefore, the OKPE-induced increase in FLG expression directly links enhanced epidermal differentiation with improved moisture retention.

The stability of tight junctions is a critical factor in fortifying the skin’s barrier and optimizing moisture retention. Claudins and occludin regulate paracellular permeability and help prevent excessive water loss [21,36]. In this study, the increased expression of claudin proteins following OKPE administration suggests a reinforcement of tight junction-mediated barrier integrity. Ceramides, the predominant lipid components of the stratum corneum, form a lamellar matrix that effectively impedes water diffusion; their depletion is a recognized hallmark of barrier impairment [23]. The elevated ceramide levels observed in this study suggest that OKPE also supports lipid-mediated barrier function. Collectively, these findings demonstrate that OKPE enhances skin barrier performance through synergistic modulation of structural scaffolds, intercellular complexes, and lipid organization.

Lastly, this study examined the impact of OKPE on the dermal layer, which provides the structural foundation for the epidermis. Collagen, the most abundant protein in the dermis, is responsible for the skin’s tensile strength and mechanical resilience. Dermal thinning and collagen fragmentation are closely associated with reduced epidermal stability and subsequent decline in skin hydration. The maintenance of collagen levels depends on the balance between TGF-β1-mediated biosynthesis and MMP-mediated degradation [37,38]. In this study, OKPE significantly enhanced TGF-β1 and type I procollagen levels while significantly suppressing MMP-1 expression. Consistently, the upregulation of COL1A1 and COL1A2 protein expression further supports the role of OKPE in maintaining collagen homeostasis. These changes indicate that OKPE contributes to the preservation of dermal structural integrity, which supports epidermal stability and hydration. Since excessive collagen degradation and dermal matrix disruption are closely associated with skin aging and loss of skin elasticity, the observed regulation of collagen synthesis protein and collagen deration protein MMP-1 expression suggests that OKPE may contribute to the maintenance of dermal homeostasis and delay of skin aging-related deterioration.

Importantly, these molecular and structural changes were reflected in functional skin outcomes. OKPE supplementation significantly reduced TEWL while increasing skin water content. These findings indicate that the improvement in skin hydration is associated with changes in HA metabolism, epidermal barrier components, and dermal structural factors observed in this study, rather than with transient surface moisturization.

The observed physiological effects are closely associated with the amino acid profile of OKPE. Leucine plays a role in epidermal homeostasis, including regulation of keratinocyte metabolism, barrier-related protein expression, and hydration-associated processes [27,39]. Its relatively high abundance in OKPE may contribute to the rapid turnover and repair of keratinocytes and dermal fibroblasts. Arginine, another major component, is a precursor for nitric oxide, which regulates blood flow and tissue repair, further supporting skin homeostasis [40,41]. In this study, amino acids identified in OKPE were categorized into BCAAs, NMF-associated, and collagen-related amino acids. BCAAs are known to support cellular homeostasis [42]. NMF-associated amino acids contribute to stratum corneum hydration through hygroscopic components such as PCA and free amino acids [34,35], whereas collagen-related amino acids serve as substrates for dermal extracellular matrix synthesis [43]. Compared with previously reported enzymatic hydrolysates derived from mackerel by-products [44], OKPE exhibited a relatively enriched composition of amino acids associated with skin hydration and dermal structural support, including leucine, glycine, and glutamic acid. These characteristics suggest the potential suitability of OKPE as a functional ingredient for skin hydration and barrier maintenance.

Collectively, these findings suggest that the balanced amino acid composition of OKPE contributes to the regulation of skin hydration, barrier function, and dermal structural integrity following oral administration. Although amino acid-based characterization was utilized in this study for quality control and functional evaluation of OKPE, further studies investigating peptide fractions and their structure–activity relationships will be valuable for identifying additional bioactive components contributing to the observed physiological effects. In addition, since only male mice were used in the present study, potential sex-related differences in skin physiological responses to OKPE could not be evaluated. Therefore, further studies including both male and female models will be necessary to better understand possible gender-dependent effects of OKPE.

## 4. Materials and Methods

### 4.1. Materials and Reagents

Frozen chum salmon heads (*O. keta*; OKH) were kindly provided by Organecotech Co., Ltd. (Seongnam-si, Republic of Korea). Protamex, a food-grade protease, was purchased from Novozymes (Bagsværd, Denmark). According to the manufacturer’s certificate of analysis, the enzyme exhibited a proteolytic activity of 1767 AU-N/g and complied with FAO/WHO JECFA and FCC specifications for food-grade enzymes. The bicinchoninic acid (BCA) protein assay kit and enhanced chemiluminescence (ECL) detection reagents were purchased from Bio-Rad (Richmond, CA, USA) and Amersham Biosciences (Piscataway, NJ, USA), respectively. Primary antibodies were purchased from Cell Signaling Technology (Danvers, MA, USA).

### 4.2. Industrial-Scale Preparation of OKPE

OKPE was obtained from MSBIO Co., Ltd. (Gimhae-si, Republic of Korea) in three independent batches (MBEL-1, MBEL-2, and MBEL-3). The preparation process is described as follows. Frozen OKH stored at 0 °C a cold-chain system was thawed for 24 h. The partially thawed OKH was soaked in running tap water at 25 °C for 1 h to remove residual blood and then minced using a food-grade dominator cutter (MSBIO Co., Ltd. (Gimhae-si, Republic of Korea)). The minced OKH (200 kg) was transferred to a 5-ton-scale reactor, and 1200 L of drinking water was added. Enzymatic hydrolysis was initiated by adding 6 kg of food-grade Protamex, and the mixture was hydrolyzed at 55 °C for 6 h. Following hydrolysis, diatomaceous earth was added as a filter aid, and the mixture was passed twice through a plate-and-frame filter press equipped with a 10 µm filter (plate size: 2000 × 2000 mm, operating pressure: 180 psi). Heat inactivation was performed at 95 °C for 10 min. The filtrate was then concentrated under vacuum conditions (−500 to −600 mmHg) at 75 °C until the soluble solid content reached ≥30 °Brix. Next, maltodextrin was added, and the mixture was thermally sterilized at 90 ± 5 °C for at least 30 min, followed by spray drying using a centrifugal atomizer with inlet and outlet air temperatures of approximately 180 °C and 85 °C, respectively. The resulting OKPE powder was stored at 26 ± 3 °C until further analysis. For biological assessments in this study, batch MBEL-1 was selected.

### 4.3. Amino Acid Profiling of OKPE

Amino acid analysis was performed using an Agilent liquid chromatography system equipped with a photodiode array detector (Agilent Technologies, Santa Clara, CA, USA). Chromatographic separation was achieved using a Capcellpak UG120 C18 column (250 mm × 4.6 mm, 5 μm). The column oven temperature was maintained at 40 °C, with an injection volume of 10 μL and a flow rate of 0.9 mL/min. The total run time was 38 min. The mobile phase consisted of solvent A (40 mM NaH_2_PO_4_, pH 7.8) and solvent B (acetonitrile:methanol:distilled water = 45:45:10, *v*/*v*/*v*). Gradient elution was performed as follows: 95% A and 5% B initially; a linear gradient to 44% A and 56% B at 31 min, maintained until 33 min; followed by a gradient to 100% B at 34 min, maintained until 38 min. Amino acids were detected at wavelengths of 338 nm and 262 nm. Based on the amino acid profile, arginine and leucine were selected as index components for OKPE quality control.

### 4.4. Animal Experiments

To evaluate the dose-dependent skin moisturizing effects of OKPE, an in vivo study was conducted using 6-week-old male ICR mice (Joongah Bio, Suwon, Republic of Korea). The animals were acclimated for one week under controlled environmental conditions (12 h light/dark cycle; 23 ± 2 °C). After acclimatization, mice were randomly assigned into five groups (*n* = 8 per group) based on body weight. OKPE was dissolved in sterile distilled water and administered orally once daily at doses of 100, 200, and 400 mg/kg for eight weeks. The 8-week experimental period was selected to allow sufficient time for physiological adaptation of epidermal barrier function, skin hydration, and dermal structural changes following long-term oral administration of OKPE. L-ascorbic acid (L-AA, 100 mg/kg/day; Sigma-Aldrich, St. Louis, MO, USA), a positive control known for its skin-protective effects in both in vitro and in vivo studies, was used for comparative evaluation. All animals were euthanized 24 h after the final administration, and skin tissue samples were collected for subsequent analyses.

### 4.5. Enzyme-Linked Immunosorbent Assay (ELISA)

Skin tissues were homogenized in ice-cold tissue lysis buffer (Invitrogen, Carlsbad, CA, USA) and centrifuged at 12,000× *g* for 15 min at 4 °C. The supernatants were collected, and total protein concentrations were determined using a BCA protein assay kit. Equal amounts of protein were used for all ELISA measurements. Skin levels of ceramide, hyaluronan, elastin, filaggrin, transforming growth factor-β1 (TGF-β1), type I procollagen, and total collagen were quantified using commercially available mouse-specific ELISA kits (AbeBio, San Diego, CA, USA), which included Mouse Ceramide ELISA Kit, Mouse Hyaluronan ELISA Kit, Mouse Elastin ELISA Kit, Mouse Filaggrin ELISA Kit, Mouse TGF-β1 ELISA Kit, Mouse Type I Procollagen ELISA Kit, and Mouse Collagen ELISA Kit. All assays were performed following the manufacturers’ instructions. Absorbance was measured at the recommended wavelength using a microplate reader, and analyte concentrations were calculated from standard curves. The results were normalized to total protein content for subsequent statistical analysis.

### 4.6. Western Blotting

Skin tissues were homogenized on ice in tissue lysis buffer (Invitrogen, Carlsbad, CA, USA) supplemented with phenylmethylsulfonyl fluoride and a protease inhibitor cocktail (Invitrogen, Carlsbad, CA, USA). The homogenates were centrifuged at 12,000× *g* for 15 min at 4 °C, and the supernatants were collected. Total protein concentrations were determined using a BCA protein assay kit (Pierce, Rockford, IL, USA). Equal amounts of protein were denatured, separated via sodium dodecyl sulfate–polyacrylamide gel electrophoresis, and transferred onto nitrocellulose membranes. The membranes were blocked with 5% nonfat dry milk for 2 h at room temperature and incubated overnight at 4 °C with primary antibodies (1:1000). After washing with Tris-buffered saline containing 0.05% Tween 20, the membranes were incubated with the appropriate horseradish peroxidase–conjugated secondary antibodies (1:3000). Protein bands were visualized using ECL reagents and quantified via densitometry using ImageJ software version 1.54 Equal amounts of total protein were loaded in each lane, and protein expression levels were normalized to glyceraldehyde-3-phosphate dehydrogenase (GAPDH), which showed stable expression across all experimental groups.

### 4.7. Measurement of Transepidermal Water Loss and Skin Water Content

TEWL and skin water content were assessed as functional indicators of skin barrier integrity and hydration status. TEWL was measured using Tewameter^®^ TM300 (Courage + Khazaka Electronic GmbH, Cologne, Germany), and skin water content was determined using a Corneometer^®^ CM825 (Courage + Khazaka Electronic GmbH, Cologne, Germany), which estimates stratum corneum hydration based on capacitance. Measurements were obtained from predefined dorsal skin regions, with three repeated readings per animal, and averaged for statistical analyses.

### 4.8. Statistical Analysis

All data are expressed as mean ± standard deviation (SD). Statistical analyses were performed using GraphPad Prism software (version 8.0.1; GraphPad Software, San Diego, CA, USA) Intergroup differences were analyzed using one-way analysis of variance, followed by Dunnett’s multiple comparisons test to compare each treatment group with the control. A *p* value < 0.05 was considered statistically significant.

## 5. Conclusions

In conclusion, this study demonstrates that oral administration of OKPE has beneficial effects on skin health. OKPE enhanced skin moisture homeostasis by regulating hydration-related factors, including HA metabolism and epidermal water transport, reinforcing epidermal differentiation, lipid barrier components, and dermal structural integrity. Importantly, the observed results were reflected in the integrated regulation of multiple epidermal and dermal factors that collectively support skin barrier stability and sustained hydration. These findings support a complementary strategy for conventional topical approaches. Furthermore, the utilization of salmon processing byproducts as a source of functional ingredients is a value-added and sustainable approach that addresses environmental concerns associated with food waste and improves resource efficiency within a circular bioeconomy framework.

## Data Availability

The data are available from the corresponding author on reasonable request.

## References

[1] Naylor R.L., Kishore A., Sumaila U.R., Issifu I., Hunter B.P., Belton B., Bush S.R., Cao L., Gelcich S., Gephart J.A. (2021). Blue food demand across geographic and temporal scales. Nat. Commun..

[2] Prabhakar P.K., Vatsa S., Srivastav P.P., Pathak S.S. (2020). A comprehensive review on freshness of fish and assessment: Analytical methods and recent innovations. Food Res. Int..

[3] Rustad T., Storrø I., Slizyte R. (2011). Possibilities for the utilisation of marine by-products. Int. J. Food Sci. Technol..

[4] Kim D.Y., Lee J.S. (2015). Directions for eco-friendly utilization and industrialization of fishery by-products. J. Fish. Mar. Sci. Educ..

[5] Atef M., Ojagh S.M. (2017). Health benefits and food applications of bioactive compounds from fish byproducts: A review. J. Funct. Foods.

[6] Radziemska M., Vaverková M.D., Adamcová D., Brtnický M., Mazur Z. (2019). Valorization of fish waste compost as a fertilizer for agricultural use. Waste Biomass Valor..

[7] Coppola D., Lauritano C., Esposito F.P., Riccio G., Rizzo C., de Pascale D. (2021). Fish waste: From problem to valuable resource. Mar. Drugs.

[8] Pandey R., Asche F., Misund B., Nygaard R., Adewumi O.M., Straume H.M., Zhang D. (2023). Production growth, company size, and concentration: The case of salmon. Aquaculture.

[9] Kandyliari A., Mallouchos A., Papandroulakis N., Golla J.P., Lam T.T., Sakellari A., Karavoltsos S., Vasiliou V., Kapsokefalou M. (2020). Nutrient composition and fatty acid and protein profiles of selected fish by-products. Foods.

[10] Im S.T., Kim W.-C., Lee S.-H. (2025). Photoprotective effect of taurine-rich Protamex extract from salmon (*Oncorhynchus keta*) byproduct against ultraviolet B-induced skin damage. Photodermatol. Photoimmunol. Photomed..

[11] Liang J., Pei X., Zhang Z., Wang N., Wang J., Li Y. (2010). The protective effects of long-term oral administration of marine collagen hydrolysate from chum salmon on collagen matrix homeostasis in aged skin. J. Food Sci..

[12] Proksch E., Fölster-Holst R., Jensen J.M. (2006). Skin barrier function, epidermal proliferation and differentiation in eczema. J. Dermatol. Sci..

[13] Rawlings A.V., Harding C.R. (2004). Moisturization and skin barrier function. Dermatol. Ther..

[14] Elias P.M. (1983). Epidermal lipids, barrier function, and desquamation. J. Investig. Dermatol..

[15] Proksch E., Brandner J.M., Jensen J.M. (2008). The skin: An indispensable barrier. Exp. Dermatol..

[16] von Stülpnagel C.C., Augustin M., da Silva N., Schmidt L., Nippel G., Sommer R. (2022). Exploring the burden of xerosis cutis and the impact of dermatological skin care from patient’s perspective. J. Dermatol. Treat..

[17] Mishra D.K., Pandey V., Maheshwari R., Ghode P., Tekade R.K. (2019). Cutaneous and transdermal drug delivery: Techniques and delivery systems. Basic Fundamentals of Drug Delivery.

[18] Crowther J.M., Sieg A., Blenkiron P., Marcott C., Matts P.J., Kaczvinsky J.R., Rawlings A.V. (2008). Measuring the effects of topical moisturizers on changes in stratum corneum thickness, water gradients and hydration in vivo. Br. J. Dermatol..

[19] Park M.-J., Song N.-E., Song H.-A., Chung K.-S., An H.-J., Hong H.-D., Choi S.Y., Rhee Y.K., Lee K.-T. (2025). Standardized hot water extract from the aerial parts of *Cirsium setidens* alleviates UVB-induced skin photoaging through inhibition of MMP-1/3 and MAPK/AP-1 pathways. J. Funct. Foods.

[20] Hyun J., Rho Y., Nagahawatta D.P., Lee G., Lee S., Ryu B., Jeon Y.-J. (2025). Upcycling fish byproducts for skin health: An enzymatic approach to sustainable nutricosmetics. Food Biosci..

[21] Yokouchi M., Kubo A. (2018). Maintenance of tight junction barrier integrity in cell turnover and skin diseases. Exp. Dermatol..

[22] Ishitsuka Y., Roop D.R. (2020). Loricrin: Past, present, and future. Int. J. Mol. Sci..

[23] Schild J., Kalvodová A., Zbytovská J., Farwick M., Pyko C. (2024). The role of ceramides in skin barrier function and the importance of their correct formulation for skincare applications. Int. J. Cosmet. Sci..

[24] Lei T., Ye L., Pei Y., Sun H., Guo C. (2025). Applications of elastin in cosmetics: Prospects and challenges. Cosmetics.

[25] Lawton S. (2019). Skin 1: The structure and functions of the skin. Nurs. Times.

[26] Alanen E., Nuutinen J., Nicklén K., Lahtinen T., Mönkkönen J. (2004). Measurement of hydration in the stratum corneum with the MoistureMeter and comparison with the Corneometer. Skin Res. Technol..

[27] Zhang X.J., Chinkes D.L., Wolfe R.R. (2004). Leucine supplementation has an anabolic effect on proteins in rabbit skin wound and muscle. J. Nutr..

[28] Dolivo D., Rodrigues A., Sun L., Galiano R., Mustoe T., Hong S.J. (2022). Reduced hydration regulates pro-inflammatory cytokines via CD14 in barrier function-impaired skin. Biochim. Biophys. Acta Mol. Basis Dis..

[29] Smith M.M., Melrose J. (2015). Proteoglycans in normal and healing skin. Adv. Wound Care.

[30] Iaconisi G.N., Lunetti P., Gallo N., Cappello A.R., Fiermonte G., Dolce V., Capobianco L. (2023). Hyaluronic acid: A powerful biomolecule with wide-ranging applications: A comprehensive review. Int. J. Mol. Sci..

[31] Fallacara A., Baldini E., Manfredini S., Vertuani S. (2018). Hyaluronic acid in the third millennium. Polymers.

[32] Karimi N., Ahmadi V. (2024). Aquaporin channels in skin physiology and aging pathophysiology. Biology.

[33] Lee S.H., Jeong S.K., Ahn S.K. (2006). An update of the defensive barrier function of skin. Yonsei Med. J..

[34] Barrett J.G., Scott I.R. (1983). Pyrrolidone carboxylic acid synthesis in epidermis. J. Investig. Dermatol..

[35] Verdier-Sévrain S., Bonté F. (2007). Skin hydration: A review on its molecular mechanisms. J. Cosmet. Dermatol..

[36] Bergmann S., von Buenau B., Vidal-y-Sy S., Haftek M., Wladykowski E., Houdek P., Lezius S., Duplan H., Bäsler K., Dähnhardt-Pfeiffer S. (2020). Claudin-1 decrease impacts epidermal barrier function in atopic dermatitis lesions dose-dependently. Sci. Rep..

[37] Shin J.-W., Kwon S.-H., Choi J.-Y., Na J.-I., Huh C.-H., Choi H.-R., Park K.-C. (2019). Molecular mechanisms of dermal aging and antiaging approaches. Int. J. Mol. Sci..

[38] Purohit T., He T., Qin Z., Li T., Fisher G.J., Yan Y., Quan T. (2016). Smad3-dependent regulation of type I collagen in human dermal fibroblasts. J. Dermatol. Sci..

[39] Collier A.E., Wek R.C., Spandau D.F. (2017). Human keratinocyte differentiation requires translational control by the eIF2α kinase GCN2. J. Investig. Dermatol..

[40] Luiking Y.C., Ten Have G.A.M., Wolfe R.R., Deutz N.E.P. (2012). Arginine de novo and nitric oxide production in disease states. Am. J. Physiol. Endocrinol. Metab..

[41] Man M.-Q., Wakefield J.S., Mauro T.M., Elias P.M. (2022). Role of nitric oxide in regulating epidermal permeability barrier function. Exp. Dermatol..

[42] Lynch C.J., Adams S.H. (2014). Branched-chain amino acids in metabolic signalling and insulin resistance. Nat. Rev. Endocrinol..

[43] de Paz-Lugo P., Lupiáñez J.A., Sicilia J., Meléndez-Hevia E. (2023). Control analysis of collagen synthesis by glycine, proline and lysine in bovine chondrocytes in vitro. Biosystems.

[44] Ramakrishnan V., Ghaly A.E., Brooks M.S., Budge S.M. (2013). Enzymatic extraction of amino acids from fish waste for possible use as a substrate for production of jadomycin. Enzyme Eng..

